# Comparative analysis of four terpenoids in root and cortex of *Tripterygium wilfordii* Radix by different drying methods

**DOI:** 10.1186/s12906-016-1453-x

**Published:** 2016-11-23

**Authors:** Tuanjie Wang, Fei Shen, Shulan Su, Yongliang Bai, Sheng Guo, Hui Yan, Tao Ji, Yanyan Wang, Dawei Qian, Jin-ao Duan

**Affiliations:** 1 Jiangsu Collaborative Innovation Center of Chinese Medicinal Resources Industrialization, Nanjing University of Chinese Medicine, Nanjing, 210023 People’s Republic of China; 2 State Key Laboratory of New-tech for Chinese Medicine Pharmaceutical Process, Jiangsu Kanion Parmaceutical CO. LTD, Lianyungang, 222001 Jiangsu China; 3 Lianyungang TCM Branch of Jiangsu Union Technical Institute, Lianyungang, 222006 China

**Keywords:** *Tripterygium wilfordii* Radix, Terpenoids, HPLC-PDA analysis, Drying temperature

## Abstract

**Background:**

*Tripterygium wilfordii* Radix, a well-known traditional medicine in china which is used for treatment of inflammation, pain, tumor and immune regulation for centuries in china, accompany with the serious toxic side effects. This study was carried out for simultaneously analyzing the four main components (triptolide, triptophenolide, demethylzeylasteral and celastrol) in *Tripterygium wilfordii* Radix under different drying processes, which was important for reducing the toxicity and quality control of *Tripterygium wilfordii* Radix in future.

**Methods:**

The terpenes were extracted by using ultrasonic method with ethyl acetate from root or cortex of *Tripterygium wilfordii* Radix, and the sensitive and rapid HPLC-PDA method was developed for simultaneous quantification of triptolide, triptophenolide, demethylzeylasteral and celastrol in root and cortex of *Tripterygium wilfordii* Radix for evaluation of the impacts by different drying processes.

**Results:**

The four compounds in their respective determined arrange had good linearity of 0.9998≦R^2^≦0.9999 and the average recoveries were range from 94.69 to 100.28%, RSDs were within 0.27 to 2.42%, respectively. The contents of triptolide, triptophenolide, demethylzeylasteral and celastrol in different *Tripterygium wilfordii* Radix individuals were varied greatly at different drying temperatures. Under different temperatures, the contents of triptolide, triptophenolide, demethylzeylasteral, and celastrol were 37.94–70.31 mg/g, 0–1.807 mg/g, 0.3513–9.205 mg/g, 3.202–15.31 mg/g, respectively. The suitable drying temperature of terpenoids in root of wild and cultivate are 80 °C and 60 °C, the suitable drying temperature of terpenoids in cortex is 40 °C.

**Conclusions:**

The method established is high sensitivity, accuracy, reliability and suitable for the simultaneous analysis of terpenoids in *Tripterygium wilfordii* Radix. The data provide a scientific basis and reference for the quality control of herb and preparations related to *Tripterygium wilfordii* Radix.

## Background

*Tripterygium wilfordii* Hook. f. and *Tripterygium hypoglaucum* (Levl.) Hutch both belong to *Tripterygium* genus of Celastraceous, their roots were considered as Lei Gong Teng (*Tripterygium wilfordii* Radix) applying to clinic in China. Lei Gong Teng shows the therapeutic effects of dispel wind and eliminate dampness, activating meridians to stop pain, detoxification and insecticide, reducing swelling, and so on [1]. In China, there are three species in this genus which mainly produced in Zhejiang, Hunan, Anhui, Fujian, Taiwan, Jilin and other provinces [2], and mainly distributed in the middle and lower reaches of Yangtze River. Currently, in clinical, the preparations of *Tripterygium wilfordii* Radix are in the form of Tripterygium Glycosides Tablet, Tripterygium total terpenoids tablets, Tripterygium bilayer tablets, Tripterygium tablets, and triptolide ointment [3], which are of great application value. So far about 100 kinds of compounds have been isolated from plants of *Tripterygium* genus [4], mainly including alkaloids, diterpenes, triterpenes, etc, which exhibited anticancer, antirheumatoid arthritis, insecticide, anti-HIV activities and were used in the treatment of kidney diseases [5–11]. The previous studies have reported that the triptolide and triptophenolide, on behalf of diterpenoid constituents, possess anti-inflammatory, immunosuppressive [12–15], antifertility, anticyst and antitumor activities [16], and so on. The demethylzeylasteral and celastrol are the major effective ingredients of triterpenoids and have a remarkable immune inhibitory activity, anti-inflammatory, anti-tumor and other pharmacological activities [17].

It was reported that *Tripterygium wilfordii* Radix has serious toxicity, and the toxic side effects incidence was 58.1% [18], which has been one of the largest herbal poisoning incidents reported in nearly half a century. Triptolide, the bioactive ingredient contained in *Tripterygium wilfordii* Radix, is also the major toxic component [19] and has a strong toxicity [20]. The roots of *Tripterygium regelii* and *Tripterygium hypoglaucum* contain similar chemical compositions [21] and have similar clinical efficacy compared with *Tripterygium wilfordii* Hook.f.. The preparations of *Tripterygium wilfordii* Radix with therapeutic doses are close to the toxic dose. Excessive doses can easily reduce reproductive system [22], urinary system [23], digestive system, nervous system [24], cortex and mucous membranes, endocrine system [25], blood system [26, 27] and other diseases, such as diarrhea, acute renal failure [28], loss of libido, vision loss, erythema, neutropenia [29], drug-induced liver injury [30], even death. So, it is important to evaluate the bioactive terpenoids and control of the quality of herbs. The multiple factors of drying processing methods, origin and breed, and different habitats for *Tripterygium wilfordii* Radix all determine the contents of bioactive terpenoids and influence the toxicity and efficacy. Therefore, it is significant for evaluating the effective and toxic components changes in *Tripterygium wilfordii* Radix during the different drying processing methods.

In this paper, a High Performance Liquid Chromatography-Photo Diode Array (HPLC-PDA) method was established to simultaneously analyze the triptolide, triptophenolide, demethylzeylasteral and celastrol and evaluate the quality of *Tripterygium wilfordii* Radix by different drying processes. It would be helpful for quality control, warranty of safety and efficacy of *Tripterygium wilfordii* Radix herb or its preparations.

## Methods

### Chemicals, reagents and materials

**Fig. 1:**
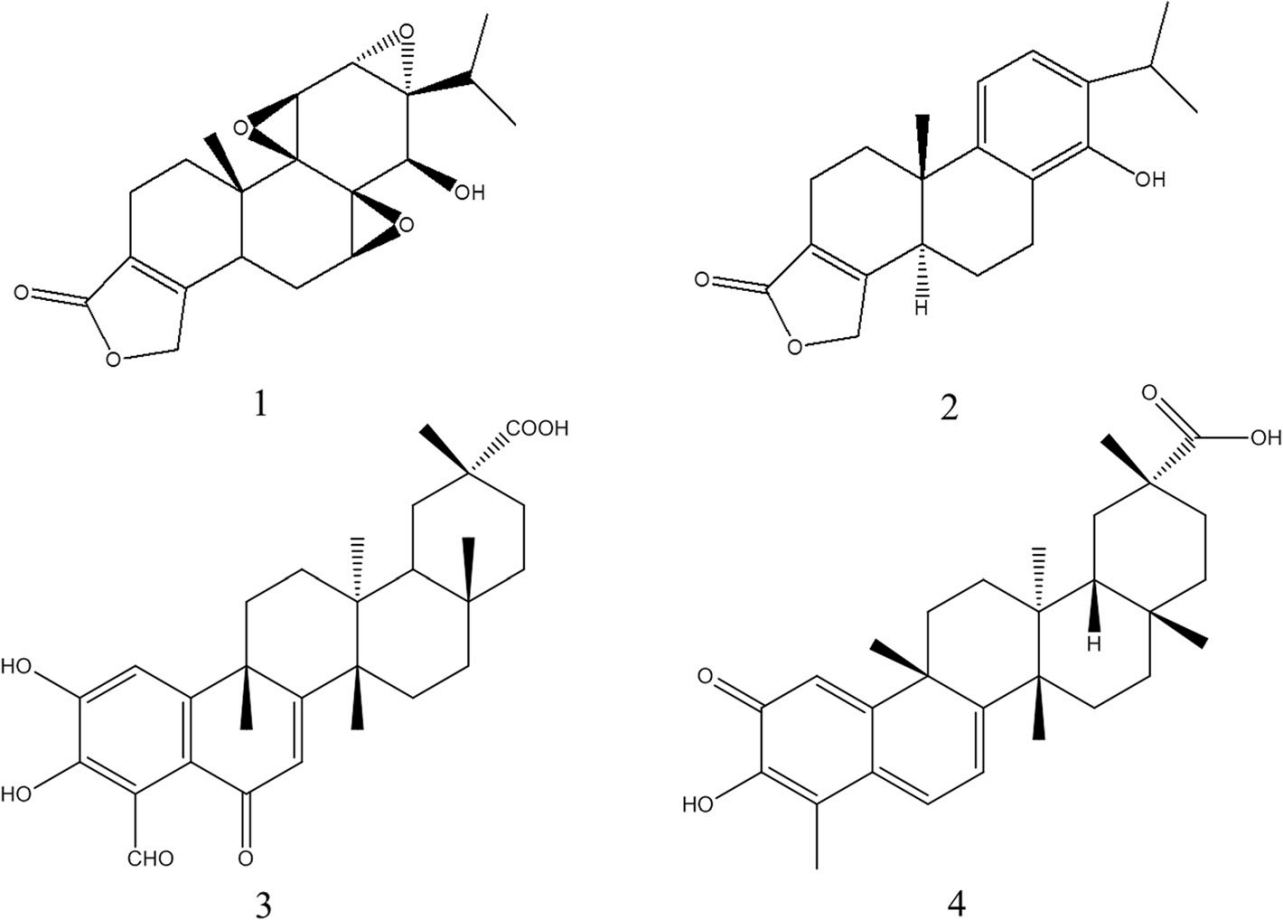
Chemical structures of four terpene compounds (1. Triptolide; 2. Triptophenolide; 3. Demethylzeylasteral; 4. Celastrol)

The reference standards of tiptophenolide (20120425) and demethylzeylasteral (ZJ0619BC13) were purchased from the Shanghai yuanye Bio-Technology Co., Ltd (Shanghai, China). The reference standards of trtiptolide (LGT-B-20131121) and celastrol (LGT-H-20121211) were purchased from the Nanjing Spring & Autumn Biological Engineering Co., Ltd (Nanjing, China). The chemical structures of them are shown in Fig. 1.

Acetonitrile and methanol were of HPLC grade and obtained from Tedia (Fairfield, USA). Formic acid and ethyl acetate were of analytical grade and from Merck (Darmstadt, Germany). Deionized water was purified by the Millipore water purification system (Millipore, Milford, MA, USA) and filtered with 0.22 *μ*m membranes. All other reagents used were of analytical grade.

The 13 batches of *Tripterygium wilfordii* Radix were collected from different origins of the whole country. Their detailed information was listed in Table 1. The corresponding author authenticated all the raw materials, and the herbal drugs were verified according to the Chinese pharmacopoeia (Chinese pharmacopoeia, 2010). The voucher specimens were deposited at the Herbarium in Nanjing University of Chinese Medicine.

**Table 1:** Sample information of Tripterygium wilfordii Radix

Number	Sample source	Plant name	planting patterns	Collection time	Species voucher number
1	Hubei Yuncheng	*Tripterygium wilfordii*	Wild	2013.08.28	No.NJUTCM-20130828
2	Hubei Shaoyang	*T. hypoglaucum*	Wild	2013.09.02	No.NJUTCM-20130902
3	Hunan Yueyang	*T. wilfordii*	Wild	2013.08.30	No.NJUTCM-20130830
4	Hunan Yueyang	*T. wilfordii*	Cultivate	2013.08.30	No.NJUTCM-20130830
5	Fujian Sanming	*T. wilfordii*	Cultivate	2013.09.05	No.NJUTCM-20130905
6	Fujian Sanming	*T. wilfordii*	Wild	2013.09.06	No.NJUTCM-20130906
7	Guizhou Leishan	*T. hypoglaucum*	Wild	2013.09.10	No.NJUTCM-20130910
8	Guizhou Jianhe	*T. hypoglaucum*	Cultivate	2013.09.09	No.NJUTCM-20130909
9	Zhejiang Xinchang	*T. wilfordii*	Cultivate	2013.09.17	No.NJUTCM-20130917
10	Zhejiang Xinhua	*T. wilfordii*	Wild	2013.09.22	No.NJUTCM-20130922
11	Jiangxi Suizhou	*T. hypoglaucum*	Cultivate	2013.10.05	No.NJUTCM-20131005
12	Jiangxi Pinxiang	*T. wilfordii*	Cultivate	2013.10.05	No.NJUTCM-20131005
13	Yunnan Yuxi	*T. hypoglaucum*	Cultivate	2013.10.10	No.NJUTCM-20131010

### Preparation of the reference solution

Triptolide, triptophenolide, demethylzeylasteral, and celastrol were accurately weighed to formulate into 0.590, 0.109, 0.928, and 0.408 mg/mL reference solution with methanol, respectively.

### Preparation of the test solution

The *Tripterygium wilfordii* Radix fresh herbs were selected randomly and separated into cortex and root under its season, 40, 60, 80, 100 °C drying and ground into powder with 40 meshes, respectively. An aliquot (1 g) was weighed precisely and ultrasound with 40 mL ethyl acetate solution for 60 min at room temperature using an ultrasonic water bath (100 kHz), and finally made to a volume of 40 mL using ethyl acetate solution. Three replicates of the extraction process were carried out on the independent samples. The solution was centrifuged 10 min at 13,000 r/min and filtered through 0.22 *μ*m membrane prior to use. Then the 10 *μ*L aliquot was injected into the HPLC system for analysis, calculate the sample content of triptolide, triptophenolide, demethylzeylasteral and celastrol.

### Chromatographic conditions

**Fig. 2:**
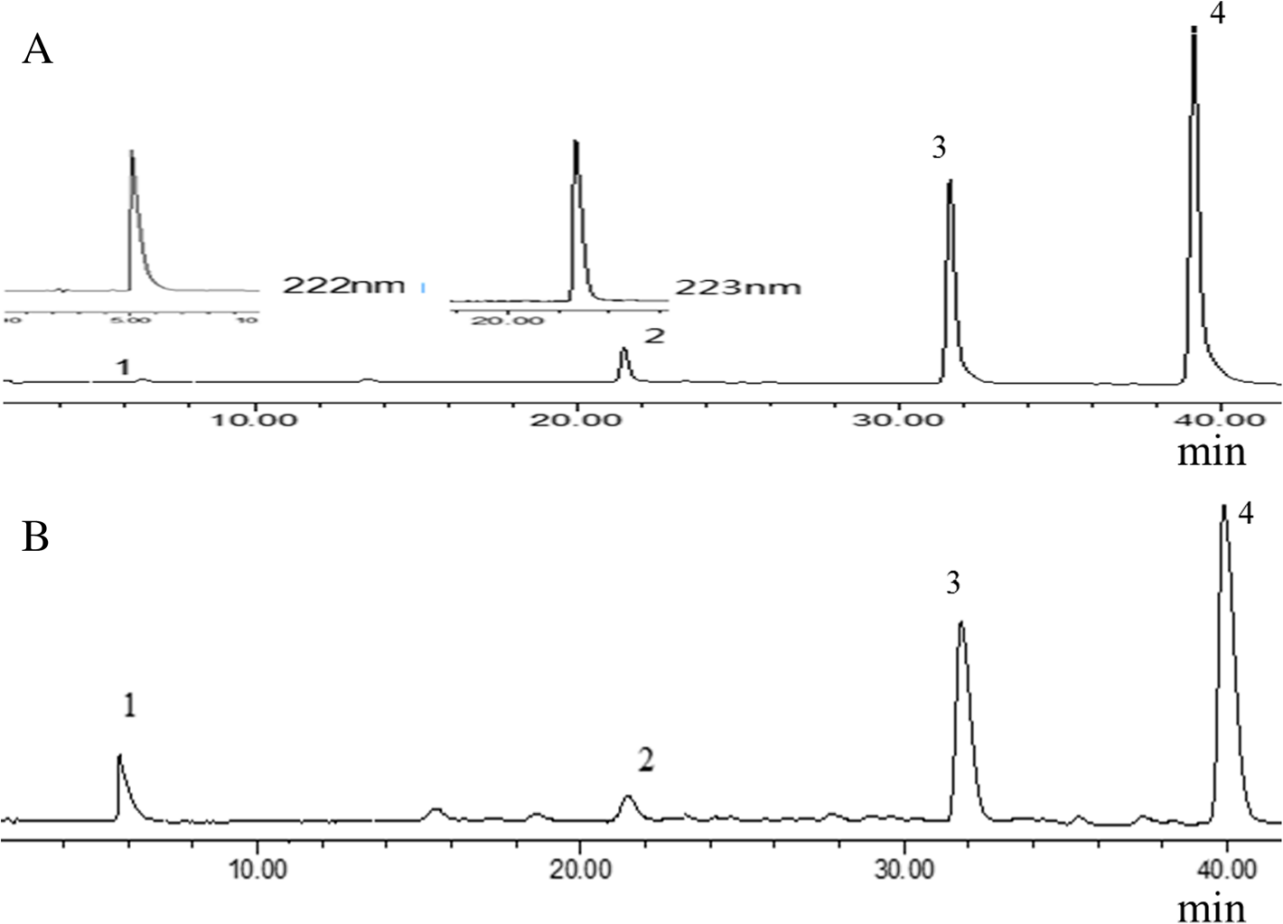
HPLC chromatograms of reference substance (**a**) and *Tripterygium wilfordii* Radix samples (**b**) (1. Triptolide; 2. Triptophenolide; 3. Demethylzeylasteral; 4. Celastrol)

The analyses were performed on a Waters 2695 Alliance HPLC system (Waters Corp., Milford, MA, USA), equipped with a quaternary pump solvent management system, an auto-sampler, and an on-line degasser. The separation was carried out on a Dimonsiol C_18_ column (250 mm × 4.6 mm, 5 *μ*m) at a column temperature of 25 °C. The mobile phase was composed of A (acetonitrile) and B (0.1% formic acid, v/v) with a gradient elution at the flow rate of 1.0 mL/min: 0 ~ 5 min, 40% A, 5 ~ 40 min, 40 ~ 90% A, 40 ~ 42 min, 90 ~ 40% A; detection wavelength was 250 nm (Diterpenoids triptolide maximum UV absorption at 222 nm, triptophenolide at maximum UV absorption at 223 nm, triterpenoids maximum absorption wavelength varied, celastrol maximum absorption at 430 nm, demethylzeylasteral maximum UV absorption at 268 nm. Considering the wavelength of maximum absorption of the compounds, the final choice was measured at 250 nm. Equilibration duration was 10 min between individual runs. A Waters 2996 photo diode array (PDA) was connected to the liquid chromatography for detection of the raw data. The HPLC chromatograms of reference substance and samples were stated in Fig. 2.

### The linear range of inspection

Take appropriate triptolide, triptophenolide, demethylzeylasteral, and celastrol reference substance to injection and record peak area values with described chromatographic conditions. A peak area values for the vertical axis, sample concentration C as abscissa. The standard stock and working solutions were all stored at 4 °C until analysis and filtered through a 0.22 *μ*m membrane prior to injection.

### Validation of the method

A series of concentrations of standard solution were prepared for the establishment of calibration curves. The peak areas were plotted against the corresponding concentrations to obtain the calibration curves. LODs and LOQs were determined using diluted standard solution when the signal-to-noise ratios (S/N) of analytes were about 3 and 10, respectively. The S/N was calculated as the peak height divided by the background noise value.

Intra-day and inter-day variations were chosen to determine the precision of the developed method. In intra-day variability test, the mixed standards solutions were analyzed for six replicates within a day; while in inter-day variability test, the solutions were examined in duplicates for consecutive three days. Repeatability was confirmed with six independent analytical sample solutions prepared from the same batch of sample. One of the sample solutions was stored at 20 °C and analyzed at 0, 2, 4, 8, 12, and 24 h, respectively, to evaluate their stability. All these variations were expressed by relative standard deviation (RSD).

A recovery test was used to evaluate the accuracy of this method. The test was performed by adding known amounts of the standards at low (80% of the known amounts), medium (the same as the known amounts) and high (120% of the known amounts) levels. The spiked samples were then extracted, processed, and quantified in accordance with the aforementioned methods. The average recovery percentage was calculated by the formula: Recovery (%) = (observed amount-original amount)/spiked amount × 100%.

## Results and discussion

### Optimization of experimental conditions

The tripterines containing carboxylic acid groups, so using a mobile phase system of aqueous acid can reduce tailing. By comparing with the acetonitrile-acid elution, both acetonitrile-0.2% phosphoric acid and acetonitrile-0.1% formic acid water solution can achieve a better separation, but phosphoric acid has some damage to column, and could easily lead to blockage of the valve, so we choose acetonitrile-0.1% formic acid as the mobile phase.

**Fig. 3:**
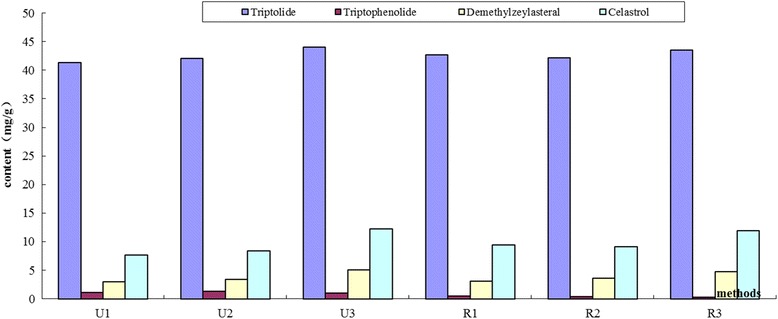
Extraction efficiency of terpenoids in *Tripterygium wilfordii* Radix with different extraction solvent (U1: ultrasonic-methanol, U2: ultrasonic-ethanol, U3: ultrasonic-ethyl acetate, R1: reflux-methanol, R2: reflux-ethanol, R3: reflux-ethyl acetate)

This experiment compared the ultrasonic-methanol (U1), ultrasonic-ethanol (U2), ultrasonic-ethyl acetate (U3), reflux-methanol (R1), reflux-ethanol (R2), reflux-ethyl acetate (R3) solvent extraction efficiency of terpenoids in *Tripterygium wilfordii* Radix. The results showed that there was a lower dissolution rate of terpenoids obtained by methanol, ethanol solvent extraction, but the acetate extraction rate is higher than that of methanol and ethanol. Besides, the dissolution rate of triptolide, demethylzeylasteral and celastrol had no significant difference between ultrasonic and reflux extraction, while the dissolution rate of triptophenolide with ultrasonic extraction was higher than reflux. Therefore, we selected the ultrasonic-ethyl acetate as the extraction solvent. The results were shown in Fig. 3.

### Methodological data results

Linear regression data, precision, repeatability, stability and recovery of 4 analytes were shown in Table 2. The results showed that the RSDs of precision test for triptolide, triptophenolide, demethylzeylasteral and celastrol were 2.95, 3.55, 0.350 and 0.270%, respectively, which indicated a good precision of the experimental method. The RSDs of stability test were 2.53, 3.04, 0.521 and 0.861%, respectively, and the absorbance of the sample solution showed stable within 24 h. The RSDs of repeatability test were 2.83, 3.40, 0.693 and 0.752%, respectively, which indicated a good reproducibility of the samples.

**Table 2:** Linear regression data, precision, repeatability, stability and recovery of 4 analytes

Analytes	Linear regression	Linear range (μg/mL)	R^2^	Precision (RSD,%)		Repeatability (RSD,%,*n* = 6)	Stability (RSD,%,*n* = 6)	LOD (ng/ml)	LOQ (ng/ml)	The average recovery (%)	
				Intra-day (*n* = 6)	Inter-day (*n* = 6)					Mean	RSD
Triptolide	*Y*_*1*_ = 282544*X*_*1*_-2417	59 l.0 ~ 3540	0.9998	2.95	1.22	2.53	2.83	1.36	4.12	96.49	1.70
Triptophenolide	*Y*_*2*_ = 853507*X*_*2*_-2479	10.9 ~ 218	0.9999	3.55	4.03	3.04	3.40	0.97	2.91	100.28	2.42
Demethylzeylasteral	*Y*_*3*_ = 12756962*X*_*3*_-560868	92.8 ~ 3612	0.9998	0.35	1.01	0.52	0.69	0.82	2.45	96.61	1.78
Celastrol	*Y*_*4*_ = 12617709*X*_*4*_-804041	40.l ~ 1224	0.9999	0.27	0.642	0.86	0.75	1.67	5.01	96.95	0.27

### Determination of terpenoids contents

The contents of terpenoids in samples were shown in Tables 3, 4, 5 and 6. The contents of triptolide, triptophenolide, demethylzeylasteral and celastrol, obtained from the *Tripterygium wilfordii* Radix samples derived from different producing areas and by different drying process temperatures, exhibited obviously different. Among which the contents of triptophenolide are obvious difference at different drying temperatures. There is trace quantity of triptophenolide in some samples of *Tripterygium wilfordii* Radix. The content of tripterine has nearly two times difference and the demethylzeylasteral about 3 times.

**Table 3:** The Triptolide content in *Tripterygium wilfordii* Radix (mg/g)

Variety	Number	Season		40 °C		60 °C		80 °C		100 °C	
		Root	Cortex	Root	Cortex	Root	Cortex	Root	Cortex	Root	Cortex
Wild	1	43.10	44.10	44.53	43.96	43.64	43.84	44.13	44.64	41.34	48.01
	2	42.76	43.36	42.10	42.45	42.48	42.99	43.62	44.08	43.50	44.64
	3	45.32	47.86	43.36	48.81	44.65	48.01	45.59	49.82	44.14	49.21
	6	51.83	64.98	52.18	66.18	53.57	60.56	55.91	63.95	55.59	66.92
	10	41.31	42.04	41.30	43.31	38.67	43.18	42.30	44.96	43.05	45.16
Cultivate	4	43.15	44.85	42.11	42.70	43.44	43.55	42.83	44.45	44.05	45.53
	5	40.03	46.22	42.17	46.45	43.85	43.54	42.47	43.25	43.40	46.34
	8	43.79	43.51	43.62	43.77	41.01	44.86	43.99	44.54	44.93	45.76
	9	42.62	42.78	43.64	44.38	42.01	47.75	42.31	46.47	45.19	47.01
	11	45.37	46.35	43.90	44.78	45.93	46.55	45.47	47.66	45.55	46.49
	12	41.00	43.45	39.71	42.29	37.94	43.65	41.66	42.76	41.31	45.08
	13	40.58	40.06	38.64	41.25	39.65	39.93	41.34	42.30	43.57	44.24

**Table 4:** The triptophenolide content in *Tripterygium wilfordii* Radix (mg/g)

Variety	Number	Season		40 °C		60 °C		80 °C		100 °C	
		Root	Cortex	Root	Cortex	Root	Cortex	Root	Cortex	Root	Cortex
Wild	1	0.318	0.642	0.226	0.845	/	0.294	/	0.473	/	/
	2	0.560	1.197	0.880	1.807	0.839	1.157	0.989	1.015	0.987	1.646
	3	/	0.214	/	0.763	0.239	0.891	0.095	0.269	0.172	0.859
	6	0.641	0.832	1.245	0.950	0.618	0.975	0.658	0.939	0.907	0.912
	7	/	0.1145	0.166	0.160	0.059	/	/	0.098	/	/
	10	0.348	0.342	0.392	0.531	0.215	0.335	0.338	0.436	0.446	0.410
Cultivate	4	0.092	0.421	0.089	0.535	0.1379	0.527	0.231	0.670	0.151	0.580
	5	/	0.439	0.091	0.347	0.118	0.393	0.347	1.164	0.096	0.761
	8	0.225	0.248	0.105	0.191	0.166	0.436	0.329	0.481	0.148	0.270
	9	0.105	0.150	0.538	0.594	0.540	0.586	0.488	0.512	0.428	0.570
	11	/	0.344	0.598	0.601	0.432	0.515	0.864	0.797	0.906	0.891
	12	0.241	0.377	0.393	0.455	0.339	0.543	0.706	0.431	0.163	0.421
	13	/	/	/	/	/	/	/	/	/	/

**Table 5:** The demethylzeylasteral content in *Tripterygium wilfordii* Radix (mg/g)

Variety	Number	Season		40 °C		60 °C		80 °C		100 °C	
		Root	Cortex	Root	Cortex	Root	Cortex	Root	Cortex	Root	Cortex
Wild	1	2.554	2.691	2.860	4.531	4.273	2.923	1.352	2.556	0.832	2.911
	2	4.001	8.530	5.432	8.275	5.441	7.584	4.922	9.205	5.066	6.908
	3	2.055	3.462	2.069	1.903	2.017	3.048	1.265	3.404	1.477	2.223
	6	3.146	5.174	5.008	6.275	4.753	4.111	3.210	4.764	1.910	3.230
	7	0.560	0.658	1.116	1.290	0.777	0.895	0.826	0.841	0.351	0.446
	10	3.740	4.490	3.726	4.740	3.271	4.397	3.732	4.364	3.489	4.399
Cultivate	4	2.385	2.561	1.678	3.260	2.400	2.389	1.831	2.792	1.725	2.303
	5	2.977	5.010	5.250	8.333	7.046	7.389	4.618	4.995	5.033	6.688
	8	4.542	6.885	7.214	8.135	5.514	7.803	4.882	8.520	5.511	7.925
	9	4.150	6.401	5.111	8.106	5.452	9.112	4.849	7.210	4.230	7.722
	11	0.970	1.105	1.365	1.816	1.380	1.736	1.050	1.891	1.769	1.861
	12	2.816	3.733	2.890	3.996	3.349	3.467	3.624	3.715	3.007	4.037
	13	1.684	1.837	1.321	1.838	2.139	2.147	1.602	1.889	1.787	1.621

**Table 6:** The contents of celastrol in *Tripterygium wilfordii* Radix by different drying methods (mg/g)

Variety	Number	Season		40 °C		60 °C		80 °C		100 °C	
		Root	Cortex	Root	Cortex	Root	Cortex	Root	Cortex	Root	Cortex
Wild	1	6.952	8.843	6.301	11.02	6.053	7.890	9.067	14.50	5.242	13.270
	2	9.112	13.876	9.112	14.02	11.54	12.36	12.80	15.32	10.330	14.990
	3	3.991	6.122	3.899	4.111	4.189	5.625	4.948	5.663	3.920	4.357
	6	4.290	7.164	4.480	7.672	7.372	9.430	6.538	10.010	5.038	7.582
	7	7.633	11.21	8.525	14.250	8.454	10.360	12.97	13.69	11.530	11.690
	10	5.040	5.063	4.822	5.076	3.974	4.773	5.032	5.242	5.097	5.187
Cultivate	4	4.940	4.958	4.643	4.695	6.176	6.757	5.432	5.524	4.869	4.926
	5	4.692	6.373	7.120	8.759	7.910	8.094	6.906	7.348	6.959	7.299
	8	5.820	6.314	3.202	7.263	7.570	7.641	5.729	7.647	6.590	7.591
	9	6.017	6.816	6.599	6.992	6.400	7.588	6.109	6.594	5.656	6.910
	11	3.733	4.932	4.321	4.900	4.464	5.529	3.819	5.253	5.254	4.965
	12	4.928	5.483	5.266	6.327	5.608	6.031	5.028	5.305	5.054	5.552
	13	4.031	4.198	3.536	4.343	4.742	4.758	4.192	4.278	4.327	4.424

Note: The “/” means not detected

**Fig. 4:**
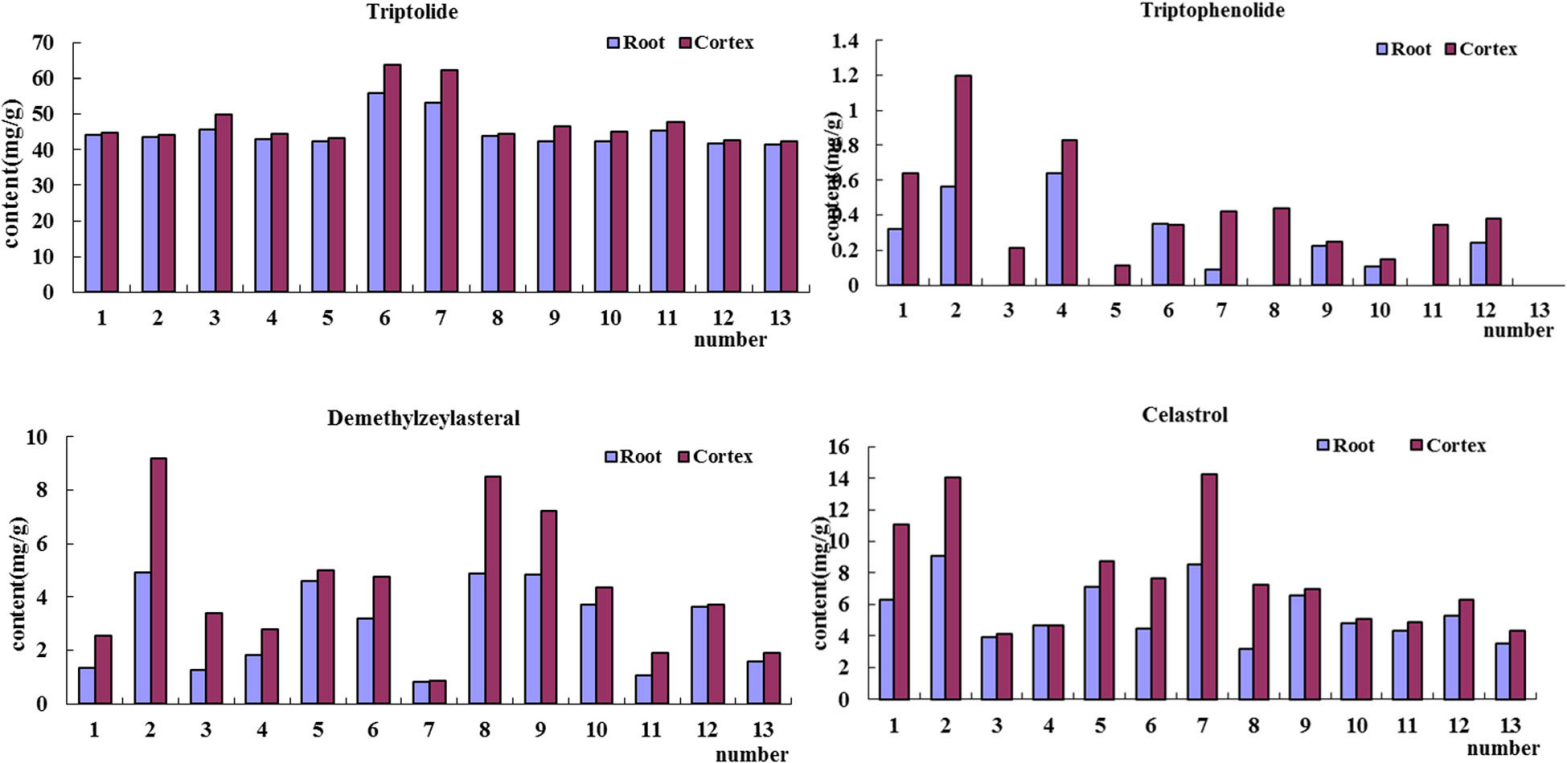
The contents of four terpenoids in cortex and root of *Tripterygium wilfordii* Radix

On the whole, the contents of terpenoids in wild *Tripterygium wilfordii* Radix are higher (The contents of triptolide, triptophenolide, demethylzeylasteral and celastrol were from 38.67 to 70.31 mg/g, 0 to 1.807 mg/g, 0.3513 to 9.205 mg/g, and 3.899 to 15.32 mg/g, respectively) than those in cultivate *Tripterygium wilfordii* Radix (The contents of triptolide, triptophenolide, demethylzeylasteral and celastrol were 37.94 ~ 47.75 mg/g, 0 ~ 1.164 mg/g, 0.9701 ~ 9.112 mg/g, and 3.202 ~ 8.759 mg/g, respectively). The content changes of terpenoids in samples from different producing areas of wild products of *Tripterygium wilfordii* Radix is relatively larger than the changes in cultivate samples*.* Besides, the contents of triptophenolide, celastrol, and demethylzeylasteral in cortex of *Tripterygium wilfordii* Radix were all higher than the contents of roots (Fig. 4) (The contents of triptolide, triptophenolide, demethylzeylasteral and celastrol were 37.94 ~ 47.37 mg/g, 0 ~ 1.164 mg/g, 0.3513 ~ 7.214 mg/g, and 3.202 ~ 12.97 mg/g, respectively) were less than the cortex part (The contents of triptolide, triptophenolide, demethylzeylasteral and celastrol were 39.93 ~ 70.31 mg/g, 0 ~ 1.807 mg/g, 0.4459 ~ 9.205 mg/g, and 4.111 ~ 15.32 mg/g, respectively).

### Compare the terpenoids in cortex of *Tripterygium wilfordii* Radix

As for the cortex of *Tripterygium wilfordii* Radix, the content of triptolide in sample from Guizhou (wild) is the highest (70.31 mg/g), Fujian (wild, 66.92 mg/g), Yueyang (wild, 49.82 mg/g) followed; the content of triptophenolide from Hubei (wild) is the highest (1.807 mg/g), Fujian (cultivate, 1.164 mg/g), Fujian (wild, 0.9746 mg/g) followed; the content of demethylzeylasteral from Hubei (wild) is the highest (9.205 mg/g), Zhejiang (cultivate, 9.112 mg/g), Guizhou (cultivate, 8.520 mg/g) followed; the content of celastrol from Hubei Shaoyang (wild) is the highest (15.32 mg/g), Hubei Yuncheng (wild, 14.50 mg/g) and Guizhou (wild, 14.25 mg/g), followed.

**Fig. 5:**
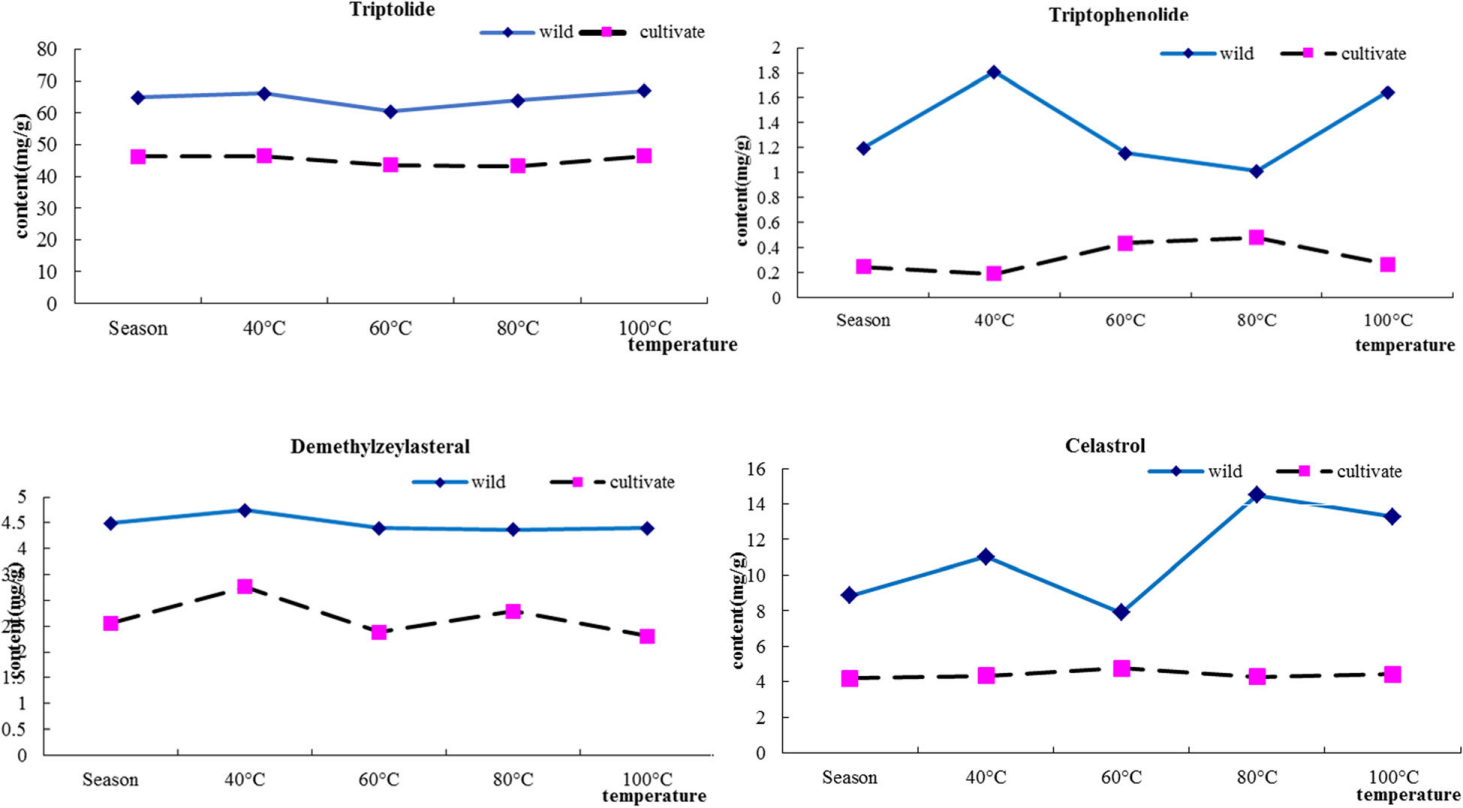
The contents of four terpenoids in wild and cultivate cortex of *Tripterygium wilfordii* Radix at different drying temperature

During the different drying temperature, the suitable drying temperature for cortex of *Tripterygium wilfordii* Radix was shown in Fig. 5. It was found that the highest content of triptolide in wild samples such as sample 1, 2, 6 and 10 were at 100 °C by comparing different temperatures. It was occupied more than half in all wild samples. The highest content of triptolide in cultivate samples such as samples 4, 8, 9, 12 and 13 were at 100 °C by comparing different temperatures. These samples are far more than in all cultivate samples in half. So it was considered that the suitable drying temperature were both 100 °C, respectively. The highest content of triptophenolide in wild samples such as sample 1, 2, 7 and 10 were at 40 °C by comparing different temperatures. The highest content of triptophenolide in cultivate samples such as samples 4, 5, 8 and 13 were at 80 °C by comparing different temperatures and they account for more than half in all wild and cultivate samples. So it was considered the suitable drying temperature were 40 and 80 °C. The highest content of demethylzeylasteral in wild samples such as sample 1, 6, 7 and 10 were at 40 °C by comparing different temperatures. It was occupied more than half in all wild samples. The highest content of demethylzeylasteral in cultivate samples such as sample 4, 5, 9, 12 and 13 were at 40 °C by comparing different temperatures. These samples are far more than in all cultivate samples in half. So it was considered the suitable drying temperature were both 40 °C. The highest content of celastrol in wild samples such as sample 1, 2, 6 and 10 were at 80 °C by comparing different temperatures. It was occupied more than half in all wild samples. The highest content of celastrol in cultivate samples such as sample 4, 5, 9, 12 and 13 were at 60 °C by comparing different temperatures. These samples are far more than in all cultivate samples in half. So it was considered the suitable drying temperature were 80 and 60 °C.

From the above conclusion, the different contents of *Tripterygium wilfordii* Radix drying temperature are different. Therefore, choose a suitable drying temperature in order to make the medicinal full and effective use is imperative. Generally speaking, the terpenoids in cortex of *Tripterygium wilfordii* Radix suitable drying temperature is 40 °C, because it could get most types of ingredients. On the contrary, it is considered the suitable drying temperature is 80 °C as for a maximum contents of total terpenoids obtained.

### Compare the terpenoids in root of *Tripterygium wilfordii* Radix

As for the root of *Tripterygium wilfordii* Radix, the content of triptolide in sample from Fujian (wild) is the highest (55.91 mg/g), Guizhou (wild, 53.30 mg/g), Jiangxi Suizhou (cultivate, 47.66 mg/g) followed. The content of triptophenolide in sample from Fujian (wild) is the highest (1.245 mg/g), Hubei Shaoyang City (wild, 0.9867 mg/g) and Jiangxi (cultivate, 0.9061 mg/g) were followed. The content of demethylzeylasteral in sample from Guizhou (cultivated) is the highest (7.214 mg/g), Fujian (cultivate, 7.046 mg/g), and Zhejiang (cultivate, 5.452 mg/g) were followed. And the content of celastrol in sample from Guizhou (wild, 12.97 mg/g) is the highest, Hubei Shaoyang (wild, 12.80 mg/g), and Hubei Yuncheng (wild, 9.067 mg/g) were followed.

**Fig. 6:**
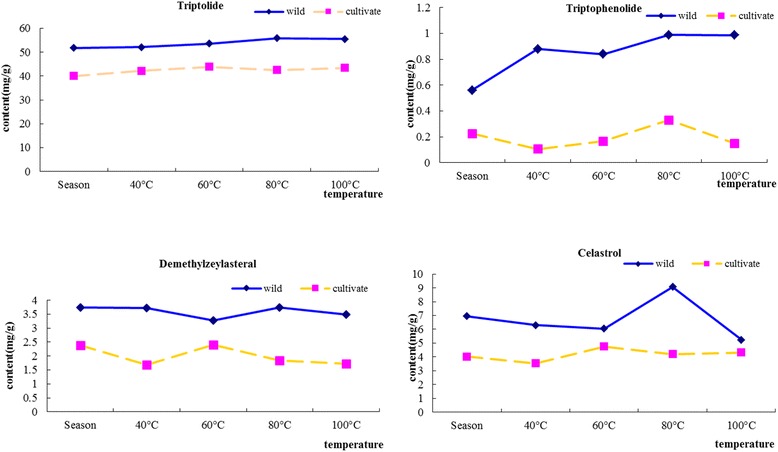
The contents of four terpenoids in wild and cultivate root of *Tripterygium wilfordii* Radix at different drying temperature

During the different drying processes, the feasible drying temperature for root of *Tripterygium wilfordii* Radix was shown in Fig. 6. It was found that the highest content of triptolide in wild samples such as sample 2, 3, 6 and 7 was at 80 °C by comparing different temperatures. The highest content of triptolide in cultivate samples such as sample 4, 8, 9 and 13 was at 100 °C by comparing different temperatures. The highest content of triptophenolide in wild samples such as sample 2, 6, 7 and 10 was at 40 °C by comparing different temperatures. The highest content of triptophenolide in cultivate samples such as sample 4, 5, 8, 11 and 12 was at 80 °C by comparing different temperatures. The highest content of demethylzeylasteral in wild samples such as sample 3, 6 and 7 was at 40 °C which account for more than half in all wild samples by comparing different temperatures. The highest content of demethylzeylasteral in cultivate samples such as sample 4, 5, 9 and 13 was at 60 °C by comparing different temperatures. The highest content of celastrol in wild samples such as sample 1, 2, 3 and 7 was at 80 °C by comparing different temperatures. The highest content of celastrol in cultivate samples such as sample 4, 5, 8, 12 and 13 was at 60 °C by comparing different temperatures.

From the above conclusion, the terpenoids in root of wild and cultivate *Tripterygium wilfordii* Radix suitable drying temperature are 80 and 60 °C, because it could get most a maximum contents of total terpenoids obtained.

## Conclusion

In this study, the HPLC-PDA assay method was established for simultaneously determination of the triptolide, triptophenolide, demethylzeylasteral and celastrol in root and cortex of *Tripterygium wilfordii* Radix by different drying processes. Compared with the previously reported methods, the present method employed a simple and rapid extraction procedure for sample preparation, and offered higher sensitivity analyzed method. The method was successfully applied to the analysis of four terpenoids in *Tripterygium wilfordii* Radix samples derived from different producing areas and different drying processes. These results would be provide an useful information for the further quality control of *Tripterygium wilfordii* Radix and its preparations and to master the dosage and indications for herbs and formulations product.

## Abbreviations

HPLC-PDA: High Performance Liquid Chromatography-Photo Diode Array
LOD: Limit of detection
LOQ: Limit of quantification
RSD: Relative standard deviation
S/N: Signal-to-noise ratios

## References

[1] State Administration of Traditional Chinese Medicine of the People’s Republic of China Chinese Materia Medica editorial board (1999). Chinese Materia Medica.

[2] Bai JP, Shi YL, Fang X, Shi Q (2003). Effects of demethylzeylasteral and celastrol on spermatogenic cell Ca^2+^ channels and progesterone induced sperm acrosome reaction. Eur J Pharmacol.

[3] Zhang SY, Shi SL (2012). Research progress on advanced pharmaceutical preparation and technology of Tripterygium and its Extracts. Strait Pharm J.

[4] Qin WZ, Lin J (2005). Advance of the research on *Tripterygium wilfordii* Hook f. to a new height. Chin J Integr Med.

[5] Shamon LA, Pezzuto JM, Graves JM, Mehta RR, Wangcharoentrakulc S, Sangsuwand R (1997). Evaluation of the mutagenic, cytotoxic, and antitumor potential of triptolide, a highly oxygenated diterpene isolated from *Tripterygium wilfordii*. Cancer Lett.

[6] Wan Y, Sun W, Zhang H, Yan QJ, Chen P, Dou CH (2010). Multi - glycoside of *Tripterygium wilfordii* Hook f. ameliorates prolonged mesangial lesions in experimental progressive glomerulonephritis. Nephron Exp Nephrol.

[7] Ji SM, Li LS, Wen JQ, Sha GZ, Cheng Z, Cheng DR (2008). Therapeutic effect of *Tripterygium wilfordii* on proteinuria associated with sirolimus in renal transplant recipients. Transplant Proc.

[8] Tao X, Fan F, Hoffmann V, Longo NS, Lipsky PE (2006). Therapeutic impact of the ethyl acetate extract of *Tripterygium wilfordii* Hook F on nephritis in NZB / W F1 mice. Arthritis Res Ther.

[9] Luo DQ, Zhang X, Tian X, Liu JK (2008). Insecticidal compounds from *Tripterygium wilfordii* active against Mythimna separata. Z Naturforsch C.

[10] Horiuch M, Murakami C, Fukamiya N, Yu D, Chen TH, Bastow KF (2006). Tripfordines A-C, sesquiterpene pyridine alkaloids from *Tripterygium wilfordii*, and structure anti-HIV activity relationships of Tripterygium alkaloids. J Nat Prod.

[11] Tao X, Wesley R, Lipsky PE (2009). Comparison of *Tripterygium wilfordii* Hook F versus sulfasalazine in the treatment of rheumatoid arthritis: a randomized trial. Ann Intern Med.

[12] Liu Y, Chen Y, Liu FQ, Lamb JR, Tam PK (2008). Combined treatment with triptolide and rapamycin prolongs graft survival in a mouse model of cardiac transplantation. Transpl Int.

[13] Xin MJ, Cui SH, Liu S, Sun HC, Li F, Sun JB (2010). Triptolide prolonged allogeneic islet graft survival in chemically induced and spontaneously diabetic mice without impairment of islet function. Hepatobiliary Pancreat Dis Int.

[14] Yang J, Xu DF, Li DZ, Lv LP, Zhang YZ, Gan M (1995). Studies on antiinflammatory and immune effects of TriptoPhenolide. Chin Tradit Herb Drug.

[15] Chen BJ (2001). Triptolide, a novel immunosuppressive and anti - inflammatory agent purified from a Chinese herb *Tripterygium wilfordii* Hook F. Leuk Lymphoma.

[16] Liu QY (2011). Triptolide and its expanding multiple pharmacological function. Int Immunopharmcol.

[17] Bian XX (2009). Advances in pharmacological effects tripterine. Chin Mod Med.

[18] Guo YH, Tan K (2007). Studies on toxicity of *Tripterygium wilfordii*. Chin Med Mat.

[19] Qian SZ, Xu Y, Zhang JW (1995). Recent progress in research on Tripterygium: a male antifertility plant. Contraception.

[20] Bai JP, Shi YL (2002). Inhibition of Ca^2+^ channels in mouse spermatogenic cells by male antifertility compounds from *Tripterygium wilfordii* Hook.f. Contraception.

[21] Si JP, Ruan XC, Guo BL, Huang WH, Xu YK. Study on the resource status and sustainable utilization of *Tripterygium wilfordii* Hook.f. Chi Tradit Herbal Drugs. 2005;28:10–1.

[22] Huang YF, Shen XS, Gu SJ, Zhu KM, Li YF (2009). Analysis on similarity of chemical compositions of between *Tripterygium wilfordii* Hook.f. and Tripterygium hypoglaucum(L · vl.) Hutchins. J Anhui Agri.

[23] Ni B, Jiang Z, Huang X, Xu F, Zhang R, Zhang Z (2008). Male reproductive toxicity and toxicokinetics of triptolide in rats. Arzneim Forsch Drug Res.

[24] Chen ZQ, Cao F, Huang HP, Huang WZ, Lin QP (2003). The effects of Tripterygium glycosides to Glomerular extracellular matrix and TGF-β1. Chin Tradit Herb Drug.

[25] Wan TJ (2005). The adverse reactions of Tripterygium. Pract Clin Med.

[26] Xue J, Jia XB, Tan XB, Chen Y, Zhang LY. Chemical constituents of *Triptergium wilfordii* Hook.f. and its toxicity. Chin J of TCM and Pharm. 2010;25:726–33.

[27] Li CX, Li TS, Zhu Z, Xie J, Lv W. Advance in studies on anti-inflammatory and immunoregulatory monomers of *Tripterygium wilfordii*. Chin J Chin Mater Med. 2014;39:4159–64. 25775786

[28] Wang Z, Jin H, Li C, Hou Y, Mei Q, Fan D (2009). Heat shock protein 72 protects kidney proximal tubule cells from injury induced by triptolide by means of activation of the MEK / ERK pathway. Int J Toxicol.

[29] Pan YQ, Gao F, Cheng J (2000). Progress of comparisive research on apoptosis and cellular nectosis. Progr Vet Med.

[30] Mei ZN, Li XK, Wu QR, Hu S, Yang XL (2005). The research on the anti-inflammatory activity and hepatotoxicity of triptolide-loaded solid lipid nanoparticle. Pharmacol Res.

